# THE OPIOID USE IN OLDER ADULTS COMPENDIUM OF RESOURCES: A USER-FRIENDLY TOOL FOR PRIMARY CARE

**DOI:** 10.1093/geroni/igae098.0204

**Published:** 2024-12-31

**Authors:** Latrice Vinson, Vonetta Dotson, Arlene Bierman

**Affiliations:** Agency for Healthcare Research and Quality, Rockville, Maryland, United States; Georgia State University, Atlanta, Georgia, United States; Georgia State University, Atlanta, Georgia, United States

## Abstract

Older adults are at greater risk of harm from opioid use, yet there is limited understanding of how to manage opioid use, misuse, and opioid use disorder among older adults, particularly in primary care practices (PCPs). AHRQ launched an initiative to synthesize the literature and create resources and tools to support PCPs in improving opioid management for older adults. This presentation describes the development of the initiative’s final product, the “2024 Opioid Use in Older Adults Compendium of Resources” and recommendations for using the tool in practice and future research. The Compendium was created though an iterative, three-stage process: environmental scan; technical expert panel feedback; and testing and refining the tool by two learning collaboratives (LCs) across 14 PCPs implementing quality improvement (QI) projects. Resources from the scan and data derived from the LCs were packaged into a driver diagram, which was iteratively revised by the panel and LCs. The resulting Compendium identified seven high-leverage changes (e.g., risk assessment, patient engagement), each associated with a change strategy, key activities, and existing and newly developed tools/resources to bring about the desired change practices can use to improve opioid management and prevent misuse in older adults. PCPs can access the user-friendly Compendium on AHRQ’s website to plan and implement QI activities related to opioid management. The Compendium identifies innovative screening measures, interventions, and patient-facing tools that present promising targets for future research.

